# Improvement of insulin sensitivity by dietary fiber consumption during late pregnant sows is associated with gut microbiota regulation of tryptophan metabolism

**DOI:** 10.1186/s42523-024-00323-6

**Published:** 2024-06-21

**Authors:** Yang Li, Jiaqi He, Lijia Zhang, Haoyu Liu, Meng Cao, Yan Lin, Shengyu Xu, Lianqiang Che, Zhengfeng Fang, Bin Feng, Jian Li, Yong Zhuo, De Wu

**Affiliations:** 1 Key Laboratory of Efficient Utilization of Non-grain Feed Resources (Co-construction by Ministry and Province), Ministry of Agriculture and Rural Affairs, College of Animal Science and Technology, Shandong Agricultural University, Panhe Street 7#, Tai’an, 271017 People’s Republic of China; 2 Key Laboratory for Animal Disease-Resistance Nutrition of the Ministry of Agriculture, Institute of Animal Nutrition, Sichuan Agricultural University, Huimin Road 211#, Chengdu, 611130 People’s Republic of China; 3 Division of Animal and Human Health Engineering, Department of Biosystems, KU Leuven, Kasteelpark Arenberg 30, Heverlee, 3001 Belgium

**Keywords:** Dietary fiber, Gut microbiota, Insulin sensitivity, Serotonin, Sow

## Abstract

**Background:**

Dietary fiber (DF) consumption was reported to improve insulin sensitivity, change the tryptophan metabolism, and alter the gut microbiota. Herein, this study aimed to investigate the effects of DF consumption on insulin sensitivity, tryptophan metabolism, and gut microbiota composition in sows during late pregnancy, and explore the relationship between tryptophan metabolites and insulin sensitivity regulated by DF supplementation.

**Results:**

Twelve sows were randomly assigned to two dietary treatment groups (six/group): the low-fiber (LF) group, which was fed a basal diet, and the high-fiber (HF) group, which was fed the basal diet supplemented with 22.60 g/kg inulin and 181.60 g/kg cellulose. During late pregnancy, meal test, glucose tolerance test, and insulin challenge test were used to investigate the insulin sensitivity of sows, using the percutaneous brachiocephalic vein catheterization technique. High DF consumption resulted in improved insulin sensitivity, especially during the second and third trimesters, and promoted serotonin production from tryptophan. Additionally, plasma serotonin concentration was positively correlated with the insulin sensitivity index during late pregnancy. Moreover, DF consumption elevated fecal short-chain fatty acid (SCFA) concentrations, altered fecal microbial diversity, and increased the abundances of *Rikenellaceae_RC9_gut_group*, *Alloprevotella*, *Parabacteroides*, *Roseburia*, and *Sphaerochaeta*, which were positively correlated to plasma serotonin concentration.

**Conclusions:**

DF consumption improved insulin sensitivity during late pregnancy in sows, which improved microbial diversity in fecal samples and increased fecal SCFA concentrations, resulting in a positive correlation with plasma serotonin level.

**Supplementary Information:**

The online version contains supplementary material available at 10.1186/s42523-024-00323-6.

## Introduction

Female hormonal, metabolic, and immunological status changes substantially during normal and healthy pregnancy [1, 2]. In mammals, insulin sensitivity increases progressively (30–70%) during the third trimester to meet the maternal metabolic needs and to provide sufficient glucose for the growth and development of the fetus [3]. Reduced insulin sensitivity or increased insulin resistance (IR) is defined as a reduced biological response of the target tissue, such as adipose tissue, liver, or muscle, to a given concentration of insulin [4]. IR is usually regarded as the primary pathological basis for reproductive dysfunction [5]. Moreover, previous studies showed that pregnant women with chronic or excessive IR are more likely to develop preeclampsia, causing short- and long-term neonatal and maternal morbidity and mortality [6, 7]. A study found that sows developed IR during late pregnancy, which was further exacerbated during lactation [8], resulting in prolonged farrowing duration, reduced lactation feed intake, and increased body weight loss during lactation, leading to a reduction in subsequent reproductive performance or even premature culling [9, 10]. Therefore, it is necessary to take measures to modulate insulin sensitivity in sows during late pregnancy.

Serotonin, an essential neurotransmitter, is a biogenic monoamine produced from tryptophan that plays an integral role in maintaining energy homeostasis and involved in numerous diseases such as gastrointestinal disorders, cardiac arrhythmia, and hypertension [11]. Watanabe et al. [12] demonstrated that increasing peripheral serotonin via intraperitoneal injection relieved high-fat diet-induced IR. Moreover, activating the serotonin receptor (5-HTR) 5-HTR_2B_ could enhance β cell proliferation and promote insulin secretion during pregnancy [13, 14]. These results highlighted the important role of serotonin in glycaemic control. It was reported that the majority (>90%) of serotonin in the body is synthesized, stored, and released from a subset of enterochromaffin cells (ECs) in the intestinal mucosa, and its biosynthesis from ECs was regulated by the gut microbiota [15]. Gut-derived serotonin can be transported to different parts of the body through serotonin transporter (SERT) and can regulate several physiological functions, including pancreatic secretion, appetite, and gastrointestinal motility [16]. Additionally, gut-derived short-chain fatty acids (SCFAs) were reported to promote the generation of colonic serotonin in colonic enterochromaffin cells (ECs) [17]. Dietary fibers (DF) are essential for human health and digestion [18]. Numerous studies have demonstrated that DF consumption can significantly reduce metabolic dysfunction and improve insulin sensitivity in mammals [19, 20], which is partially attributed to the production of SCFAs and alteration of the gut microbiota especially SCFA-producing bacteria [21]. Our recent study in sows showed that a DF-supplemented gestating diet increased colonic SCFAs (including acetate, propionate, and butyrate) and promoted colonic serotonin generation [22]. However, it remains unclear that the role of serotonin in DF-induced regulation of insulin sensitivity during pregnancy.

In view of the above, we hypothesized that DF consumption during gestating period could improve insulin sensitivity through increasing the peripheral serotonin concentration regulated by the gut microbiota in sows. Therefore, this study aimed to investigate the effects of DF consumption on insulin sensitivity, tryptophan metabolism, and gut microbiota composition in sows during the third trimester, and to explore the possible role of tryptophan metabolism in DF-induced regulation of insulin sensitivity.

## Materials and methods

### Animals, diets, and management

Twelve Large White × Landrace crossbred sows (average body weight: 132.04 ± 1.87 kg and back fat thickness: 13.96 ± 0.66 mm) were bred with Duroc boars and assigned randomly to two dietary treatment groups (six/group) after artificial insemination: low-fiber (LF) group and high-fiber (HF) group. The LF group sows were fed a basal diet [1.10% soluble fiber (SF) and 9.14% insoluble fiber (ISF)], while the HF group sows were fed a high-fiber diet (2.77% SF and 22.66% ISF) prepared from the basal diet supplemented with extra 22.60 g/kg inulin and 181.60 g/kg cellulose. Inulin and cellulose used in this study were both commercial products, and purchased from ZTH tech (Beijing, China) and Guangxi Shangda Tech Co. (Nanning, China), respectively. The purity of inulin and cellulose was >90%. The basal diet (Table 1) was formulated in compliance with National Research Council (NRC, 2012) [23] to meet the nutrient requirements of gestating sows. The meals were provided once daily at 08:00 am with *ad libitum* access to water. The daily gestation diet intake in the LF group was 2.15 kg from day 1 to 89, 2.55 kg from day 90 to 112, and 1.90 kg from day 113 to parturition; and corresponding values in the HF group were 2.59, 3.07, and 2.29 kg.

**Table 1:** Composition and calculated analysis of basal diets (as-fed basis)

Items	Basal diet^a^
**Ingredients, %**	
Corn	62.39
Dehulled soybean meal, 46%	13.10
Fish meal, 53.5%	2.00
Wheat flour	10.00
Corn starch	10.00
L-lysine HCl, 76.8%	0.10
L-threonine, 98%	0.02
Limestone	0.84
Monocalcium phosphate	0.46
Sodium chloride	0.40
Choline	0.14
Vitamin premix	0.05
Trace mineral premix	0.50
Total	100.00
**Nutrients content** ^**b**^	
Digestible energy, MJ/kg	14.06
Crude protein, %	13.39
Crude fat, %	2.90
Crude fiber, %	1.41
Soluble fiber, %	1.10
Insoluble fiber, %	9.14
Total dietary fiber, %	10.21
Ca, %	0.60
Available P, %	0.27
Lysine, %	0.60
Methionine, %	0.21
Threonine, %	0.46
Tryptophan, %	0.14

^a^Provided per kilogram of complete diet: vitamin A 7500 IU, vitamin D_3_ 5000 IU, vitamin E 37.5 IU, vitamin K_3_ 5 mg, vitamin B_1_ 5 mg, vitamin B_2_ 12.5 mg, vitamin B_6_ 7.5 mg, vitamin B_12_ 0.05 mg, biotin 0.2 mg, niacin 50 mg, folic acid 2.5 mg and D-calcium pantothenate 25 mg, 10 mg of Cu as CuSO_4_, 100 mg of Fe as FeSO_4_, 0.6 mg of I as KI, 100 mg of Zn as ZnSO_4_, 30 mg of Mn as MnSO_4_ and 0.25 mg of Se as Na_2_SeO_3_

^b^All data were calculated according to the tables of Feed Composition and Nutrient Values in China (2023) in the basal diet

### Experimental design

The meal test, glucose tolerance test (GTT), and insulin challenge test (ICT) were conducted via the percutaneous brachiocephalic vein catheterization technique to determine the effects of DF consumption on insulin sensitivity during the third trimester. At day 80 of pregnancy, a customized catheter (inner diameter: 0.96 mm and outer diameter: 1.68 mm), soaked successively in 0.6% tridodecylmethylammonium chloride (CAS#7173-54-8), 6.5% heparin sodium (CAS#9041-08-1), and 0.1% chlorhexidine acetate (CAS#56-95-1), was fixed to the left brachiocephalic vein after the sow was anesthetized by intramuscular injection of Zoletil 50 (Virbac, Carros, France) [24]. The catheter was rinsed twice daily using 2% heparin sodium to remove any obstructions.

On the morning of days 85, 97, and 110 of pregnancy, blood samples were collected 15 and 5 min before and 10, 30, 60, 90, 120, 180, and 240 min after the beginning of the meal (08:00 am, time 0) [25]. On the subsequent mornings (days 86, 98, and 111), the intravenous (i.v.) GTT was initiated at 08:00 am (time 0) and the blood samples were collected 15 min before and 5, 30, 60, 90, 120, and 180 min after the infusion of 0.5 g of glucose/kg BW (50% glucose injection; Kelun Pharmaceutical Co., Ltd., Xiantao, Hubei, China) for 5 min, through the jugular catheter [25]. Subsequently, the i.v. ICT was initiated at 2:00 pm (time 0) and the blood samples were collected 15 min before and 5, 30, 60, 90, 120, and 180 min after infusion of 0.1 U of insulin/kg BW (40 IU/mL insulin injection; Jiangsu Wanbang Biochemical Medicine Group Co., Ltd., Xuzhou, China) for 1 min, through the jugular catheter [8]. After the infusion and blood collection, 2 mL of 2% heparin sodium was injected to rinse the catheter immediately. During the test days, the sows were not fed before the i.v. GTT, but fed immediately after i.v. ICT. All the blood samples at each time point were collected in tubes containing heparin sodium after blood glucose (BG) measurement using a glucose meter (Sannuo, Changsha, China), and the obtained plasma samples were analyzed for insulin concentration. In the meal test, the insulin sensitivity index was calculated as 1/[fasting BG (FBG) × fasting insulin (FIN)] and the insulin resistance index (HOMA-IR) was calculated as FBG×FIN/22.5, in which the FBG and FIN referred to the average fasting blood glucose and fast blood insulin, respectively [26]. In addition, the glucose disposal rate was calculated by the slope of glucose change against time from 5 to 30 min after the i.v. GTT and the half-life of glucose was calculated as described previously [27].

### Sampling

On day 110 of pregnancy, fresh fecal samples were collected from the rectum of the 12 sows, before feeding in the morning, and the outermost parts and the parts against the intestinal wall were discarded. The fecal samples were then divided into two sterile tubes for the determination of SCFA concentrations and microbial composition, respectively. The fecal samples were stored at −80 °C until the analysis.

### Determination of blood glucose (BG) and insulin concentrations

The BG values were measured using the glucose meter (Sannuo), within 10 s after blood samples collection. The insulin concentration was detected by radioimmunoassay using guinea pig anti-porcine insulin serum (#R-C-02-01; 3 V Bioengineering Group Co., Ltd., Weifang, China), as described previously [28].

### Determination of plasma tryptophan, kynurenine, and serotonin concentrations

The fasting (5 min before the meal) plasma tryptophan and kynurenine concentrations of sows on day 110 of pregnancy were measured using high-performance liquid chromatography (HPLC), as described by Veit et al. [29]. The pure compounds or internal standards for HPLC were purchased from Sigma-Aldrich (Darmstadt, Germany). The preprandial and postprandial plasma serotonin concentrations were determined using a commercially available ELISA kit (#EA602/96; DLD Diagnostika GmBH, Hamburg, German), according to the manufacturer’s instructions [30].

### Determination of fecal SCFA concentrations

The fecal SCFA concentrations of sows on day 110 of pregnancy were measured using gas chromatography (Varian CP-3800 GC, United States), as described in Li et al. [31]. Briefly, the fecal sample was suspended in 1.5 mL of distilled water and the supernatant was obtained and mixed with metaphosphoric acid (CAS#37267-86-0), crotonic acid (CAS#107-93-7), and HPLC-grade methanol (CAS#67-56-1). Finally, 1 µL of the supernatant was analyzed for acetate, propionate, and butyrate concentrations, and total SCFAs was calculated as their sum.

### Microbial composition and diversity analysis

The microbial genomic DNA was extracted from frozen fecal samples of the 12 sows (6 sows per group) on d 110 of pregnancy using an E.Z.N.A. TM Stool DNA kit (#D4015-02; Omega Bio-Tek, Norcross, Georgia, USA) as described previously [32]. After DNA concentration and purity examination, the V4 hypervariable region of 16 S rDNA was amplified with the barcoded primers (515 F-806R; 5′-GTGCCAGCMGCCGCGGTAA-3′ and 5′-GGACTACHVGGGTWTCTAAT-3′, respectively) [33]. Generated sequencing libraries were sequenced on the Illumina HiSeq PE2500 platform (Novogene, Beijing, China). After paired-end reads assembly, data filtration, and chimera removal, the effective sequences were obtained, and sequences at 97% sequence similarity were clustered into the same operational taxonomic units (OTUs) using UPARSE pipeline [34]. Observed species, Simpson index (1-D form), Shannon index, Chao 1 index, and ACE index were calculated to assess the difference in alpha diversity. Bray_curtis distance and UPGMA phylogenetic tree were used for comparison of taxonomic data in beta diversity using the QIIME2 and displayed with R software (V3.1) [35, 36]. Significant differences in the microbial communities of the two groups were detected with the analysis of similarity (ANOSIM) test.

### Statistical analysis

The individual sow was considered the experimental unit for all the variables, and the SAS 9.4 ((Institute Inc., Cary, NC, USA) was used to compare the significance between LF group and HF group. Postprandial blood glucose, insulin, and serotonin concentrations were analyzed using repeated-measures, and the fasting basal values were used as a covariate. The other values were analyzed using the t-test procedure. Normality of data distribution was assessed with a Shapiro-Wilk’s statistic (*W* > 0.05). Spearman’s correlations were used to examined the associations between insulin sensitivity and plasma serotonin concentration as well as between bacterial abundance and plasma serotonin concentration. Values were expressed as mean ± standard error. The level of statistical significance was set at *P* < 0.05, and 0.05 < *P* < 0.10 was considered a trend toward significance.

## Results

### Effect of DF consumption on changes of BG and insulin concentrations during the third trimester

**Fig. 1:**
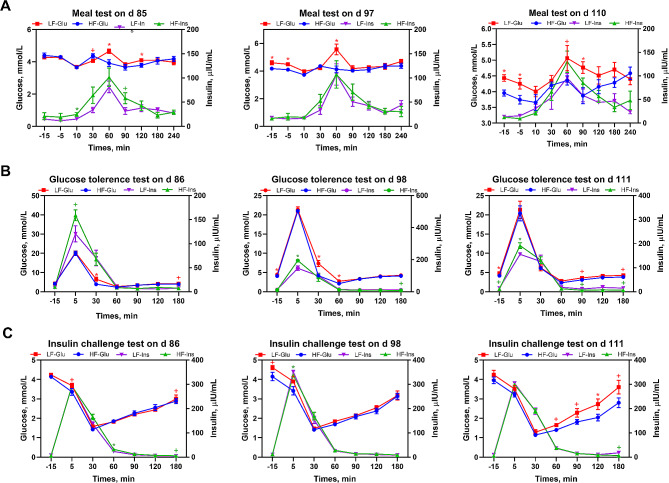
Blood glucose and insulin concentrations following the meal test, the i.v. glucose tolerance test, and the i.v. insulin challenge test. (**A**) Blood glucose and insulin concentrations before and after the morning meal on d 85, 97, and 110 of pregnancy; (**B**) Blood glucose and insulin concentrations before and after the i.v. glucose tolerance test on d 86, 98, and 111 of pregnancy; (**C**) Blood glucose and insulin concentrations before and after the i.v. insulin challenge test on d 86, 98, and 111 of pregnancy. *LF* sows fed a basal diet, *HF* sows fed with the basal diet added with extra 22.60 g/kg inulin and 181.60 g/kg cellulose. *n* = 6 per group. Values are mean ± standard error. The level of statistical significance was set by *P < 0.05 and and **P < 0.01, and ^+^0.05 < *P* < 0.10 was considered a trend toward significance

The meal test (Fig. 1A) results showed that the BG concentration decreased 10 min after the morning meal. Furthermore, on days 85 and 97 of pregnancy, the BG concentration in the LF and HF groups peaked at 60 and 30 min after the initiation of the meal, respectively. On day 110 of pregnancy, the BG concentration peaked at 60 min in both the groups. The range of glycemic fluctuations in the HF group was reduced compared with that in the LF group. The blood insulin concentration peaked 60 min after the initiation of the meal in both groups on days 85, 97, and 110 of pregnancy. On day 85 of pregnancy, the BG concentrations of the HF group were significantly lower at 60 and 120 min (*P* < 0.05) and higher at 30 min (*P* < 0.10), compared to those of the LF group. Additionally, on day 85, the HF group showed higher blood insulin concentration at 10 (*P* < 0.05) and 90 min (*P* < 0.10) compared with the LF group. Moreover, on days 97 and 110 of pregnancy, the HF group showed significantly decreased (*P* < 0.05) FBG and BG concentrations at 60 and 90 min, respectively, and decreased BG concentration (*P* < 0.10) at 60 min on day 110, compared with the LF group.

The i.v. GTT results (Fig. 1B) demonstrated that glucose injection resulted in hyperglycemia and that the BG and insulin concentrations reached the maximum values 5 min after the injection. Glycemia then decreased rapidly, especially in the HF group, leading to hypoglycemia after 60 min of glucose injection. Additionally, rapid plasma insulin release was observed in the HF group 5 min after the injection on days 86 (*P* < 0.10), 98 (*P* < 0.05), and 111 (*P* < 0.05) of pregnancy, after the initiation of the i.v. GTT. Moreover, on day 86 of pregnancy, compared with the LF group, the HF group had significantly lower (*P* < 0.05) BG concentration after 30 min and lower (*P* < 0.10) BG concentration after 180 min of glucose injection. On day 98 of pregnancy, compared with the LF group, the HF group had significantly lower (*P* < 0.05) BG concentration 15 min before and during 30–60 min of glucose injection and reduced (*P* < 0.10) plasma insulin concentration after 180 min of glucose injection. Moreover, on day 111 of pregnancy, FBG concentration decreased significantly (*P* < 0.05), while FIN, BG, and insulin concentrations deceased (*P* < 0.10) at 90 and 180 min in the HF group, compared with the LF group.

The i.v. ICT results (Fig. 1C) showed that the insulin concentration peaked after 5 min, while the BG concentration decreased after 30 min of insulin injection. On day 86 of pregnancy, the HF group showed a decrease (*P* < 0.10) in the BG concentration at 5 and 180 min and an increase in the plasma insulin concentration at 60 (*P* < 0.05) and 180 (*P* < 0.10) min, compared with the LF group. On day 98 of pregnancy, compared with the LF group, the HF group showed decreased (*P* < 0.10) BG concentrations 15 min before and 5 min after the insulin injection and significantly decreased (*P* < 0.05) plasma insulin concentration 5 min after the insulin injection. On day 111 of pregnancy, compared to the LF group, the HF group showed significantly decreased (*P* < 0.05) BG concentrations at 120 min and decreased (*P* < 0.10) BG concentrations at 60, 90, and 180 min and plasma insulin concentration at 180 min.

### Effect of DF consumption on parameters related to insulin sensitivity during the third trimester

As shown in Table 2, compared with the LF group, the HF group showed significantly increased (*P* < 0.05) insulin sensitivity indexes and significantly decreased (*P *< 0.05) HOMA-IR indexes on days 97 and 110 of pregnancy. Additionally, compared with the LF group, the HF group showed a significant increase (*P* < 0.05) in glucose disposal rate and a significant decrease (*P* < 0.05) in half-time of glucose on day 98 of pregnancy.

**Table 2:** Effects of dietary fiber supplementation on insulin sensitivity index, HOMA-IR, glucose disposal rate and half-time of glucose in gestating sows

Items	Groups	
	LF	HF
**Insulin sensitivity index**		
d 85	0.019 ± 0.002	0.022 ± 0.006
d 97	0.015 ± 0.003	0.021 ± 0.003*
d 110	0.018 ± 0.002	0.034 ± 0.005*
**HOMA-IR**		
d 85	2.55 ± 0.31	2.50 ± 0.61
d 97	3.63 ± 0.74	2.26 ± 0.29*
d 110	2.84 ± 0.55	1.43 ± 0.18*
**Glucose disposal rate**		
d 86	0.58 ± 0.03	0.63 ± 0.04
d 98	0.56 ± 0.02	0.67 ± 0.01**
d 111	0.60 ± 0.06	0.57 ± 0.08
**Half-life of glucose**		
d 86	1.21 ± 0.05	1.13 ± 0.08
d 98	1.25 ± 0.05	1.03 ± 0.01**
d 111	1.22 ± 0.12	1.34 ± 0.14

*n* = 6 per group. Values are mean ± standard error

*LF* sows fed a basal diet, *HF* sows fed with the basal diet added with extra 22.60 g/kg inulin and 181.60 g/kg cellulose

The level of statistical significance was set by *P < 0.05 and and **P < 0.01

### Effect of DF consumption on tryptophan metabolism on day 110 of pregnancy

**Fig. 2:**
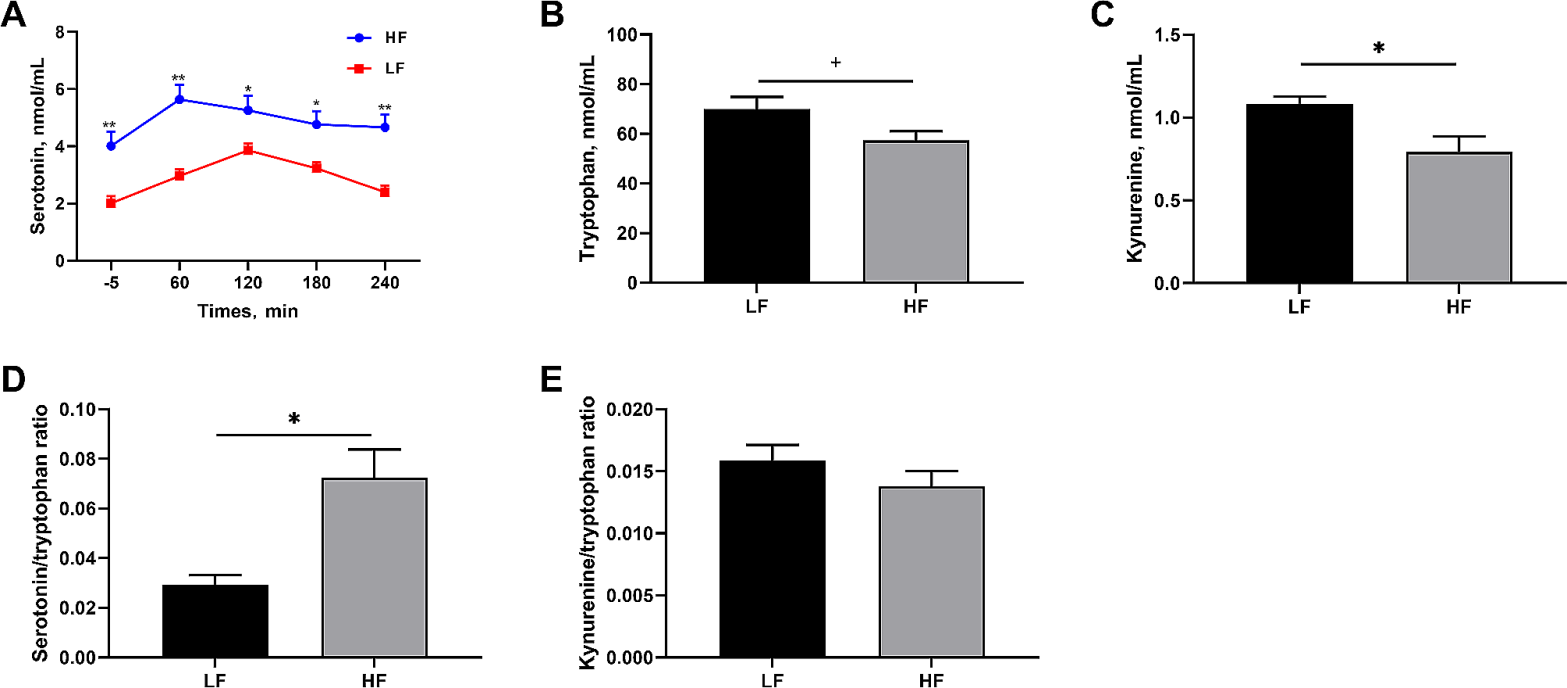
Effect of dietary fiber consumption on tryptophan metabolism on d 110 of pregnancy. (**A**) Plasma serotonin concentration before and after the morning meal; (**B**) Plasma tryptophan concentration; (**C**) Plasma kynurenine concentration; (**D**) Plasma serotonin/ tryptophan ratio; (**E**) Plasma kynurenine/ tryptophan ratio. *LF* sows fed a basal diet, *HF* sows fed with the basal diet added with extra 22.60 g/kg inulin and 181.60 g/kg cellulose. *n* = 6 per group. Values are mean ± standard error. The level of statistical significance was set by *P < 0.05 and **P < 0.01, and ^+^0.05 < *P* < 0.10 was considered a trend toward significance

As shown in Fig. 2A, after the morning meal, the plasma serotonin levels in the HF and LF groups peaked at 60 and 120 min, respectively. Additionally, compared with the LF group, the HF group showed significantly increased (*P* < 0.05) plasma serotonin concentrations 5 min before and 60, 120, 180, and 240 min after the meal. Moreover, compared with the LF group, the HF group showed significantly lower (*P* < 0.05) kynurenine (Fig. 2C) and lower (*P* < 0.10) tryptophan concentrations (Fig. 2B) and significantly higher (*P* < 0.05) serotonin/tryptophan ratio (Fig. 2D) in fasting plasma. However, there was no significant difference (*P* > 0.05) in the fasting plasma kynurenine/tryptophan ratio between the two groups (Fig. 2E).

### Effect of DF consumption on fecal SCFA concentrations on day 110 of pregnancy

The fecal SCFA concentrations on day 110 of pregnancy are shown in Table 3. The HF group showed significantly higher (*P* < 0.05) concentrations of acetate, butyrate, and total SCFAs and higher (*P* < 0.10) concentration of propionate, compared to the LF group.

**Table 3:** Effect of dietary fiber consumption on short-chain fatty acids (SCFAs) concentrations in fresh feces on d 110 of pregnancy

Items	Groups	
	LF	HF
Acetate, µmol/g	28.71 ± 2.97	40.65 ± 2.63**
Propionate, µmol/g	17.52 ± 2.45	24.94 ± 2.58^+^
Butyrate, µmol/g	9.48 ± 1.24	16.12 ± 2.11*
Total SCFAs, µmol/g	55.71 ± 4.88	81.71 ± 6.23**

*n* = 6 per group. Values are mean ± standard error. Total SCFAs = Acetate + Propionate + Butyrate

*LF* sows fed a basal diet, *HF* sows fed with the basal diet added with extra 22.60 g/kg inulin and 181.60 g/kg cellulose

The level of statistical significance was set by *P < 0.05 and **P < 0.01, and ^+^0.05 < *P* < 0.10 was considered a trend toward significance

### Effect of DF consumption on fecal microbial composition and diversity on day 110 of pregnancy

**Fig. 3:**
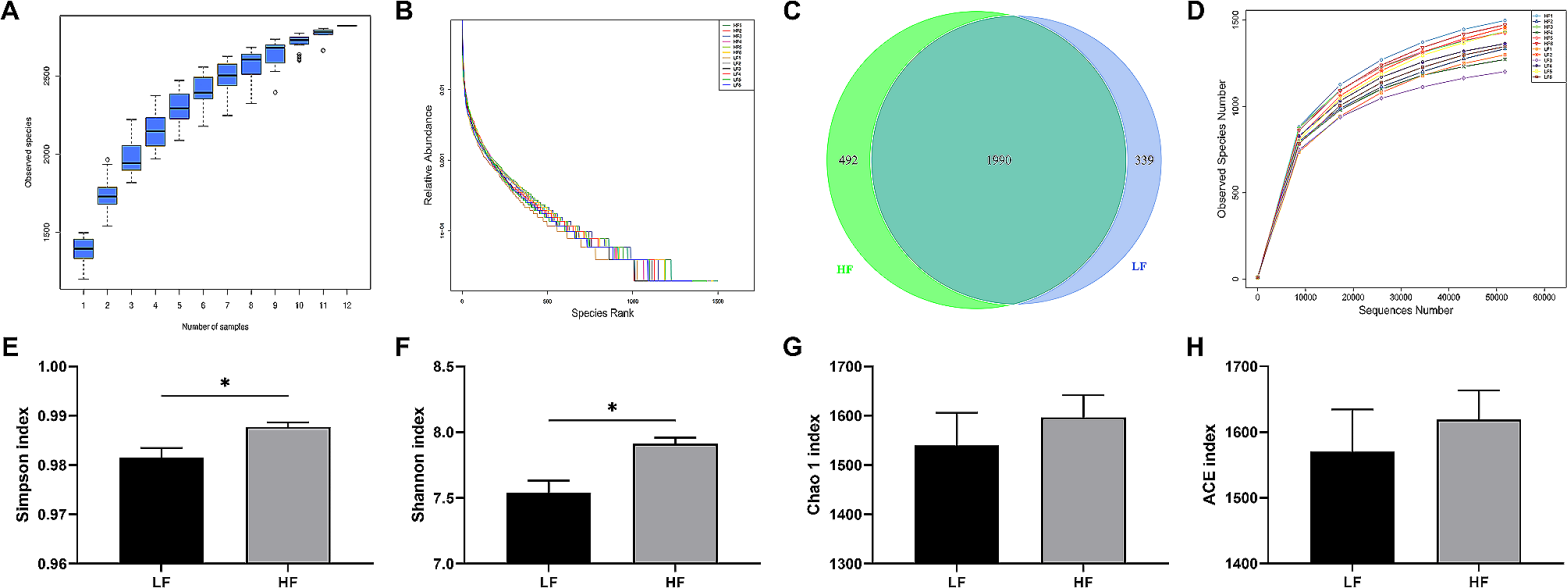
Effect of dietary fiber consumption on fecal microbial community diversity and richness on pregnancy d110. (**A**) Species accumulation curves; (**B**) Species rank curves; (**C**) Venn diagram generated to depict shared and unique sequences between the two groups; (**D**) Rarefaction curve; (**E**–**H**) Alpha diversity indexes, containing Simpson (1-D form), Shannon, Chao 1, and ACE indexes. *LF* sows fed a basal diet, *HF* sows fed with the basal diet added with extra 22.60 g/kg inulin and 181.60 g/kg cellulose. *n* = 6 per group. The level of statistical significance was set by *P < 0.05

As displayed in Fig. 3, the species accumulation curves (Fig. 3A), which present cumulative counts of species with sampling number, flattened as the number of sequences increased to 12, demonstrating that the sequencing was deep enough to cover the species richness and diversity of the samples. As seen in the species rank curve (Fig. 3B), which represents the diversity of samples within a group, the HF group had a higher species richness and a more homogeneous species distribution. The Venn diagram (Fig. 3C) also indicated that the HF group had more unique sequences than the LF group. Moreover, the Simpson (Fig. 3E) and Shannon (Fig. 3F) indexes were significantly higher (*P* < 0.05) in the HF group than in the LF group; however, no significant differences (*P* > 0.05) were noted in the observed species (Fig. 3D), Chao 1 (Fig. 3G), and ACE (Fig. 3H) indexes between the two groups.

**Fig. 4:**
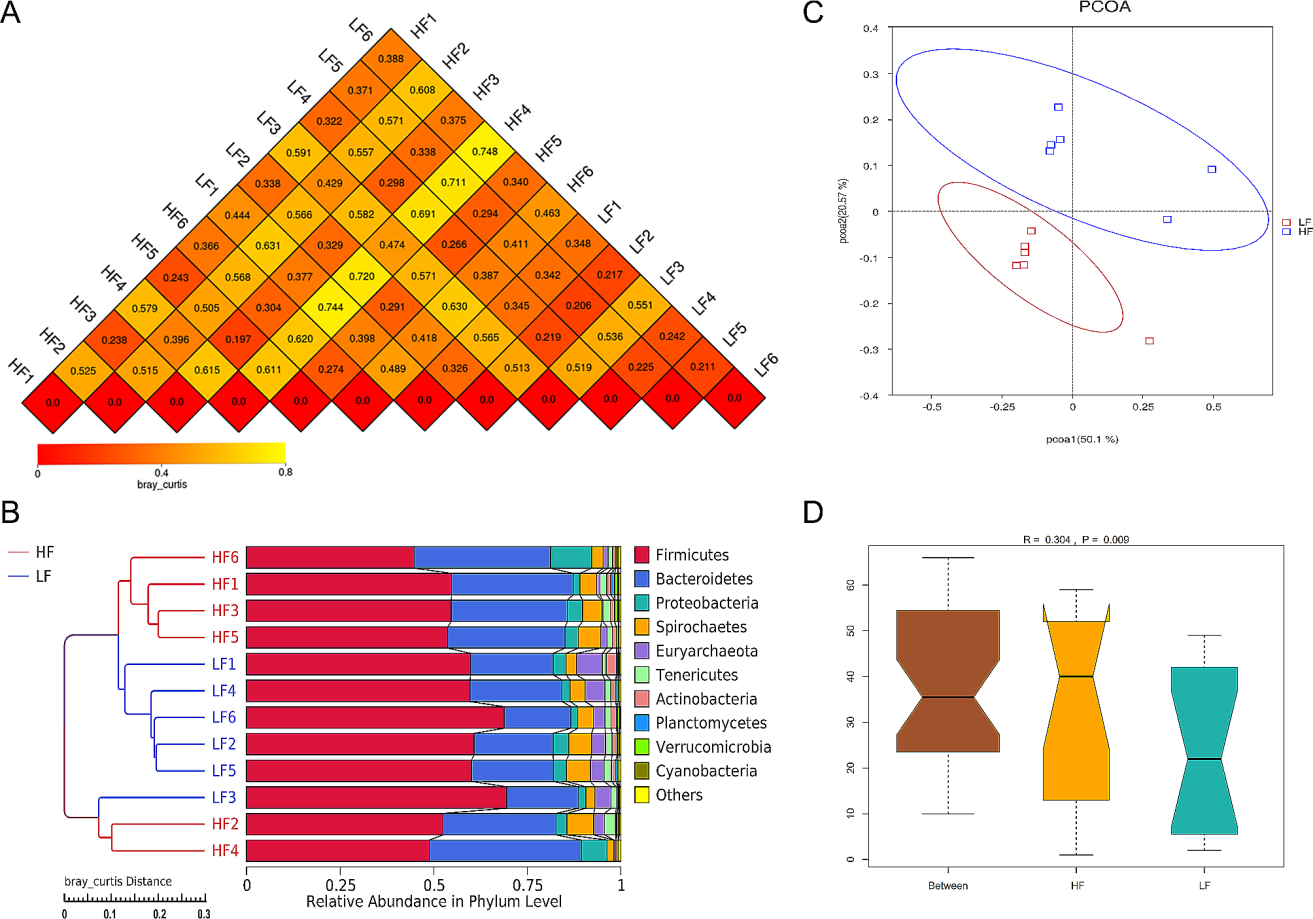
Beta diversity of fecal microbial community analysis on d 110 of pregnancy. (**A**) Heat-map of beta diversity for each two samples by bray_curtis distance; (**B**) The principal coordinate analysis (PCoA) profile of bray_curtis distance; (**C**) Unweighted pair-group method with arithmetic mean (UPGMA) clustering analysis with bray_curtis distance; (**D**) Analysis of ANOSIM. *LF* sows fed a basal diet, *HF* sows fed with the basal diet added with extra 22.60 g/kg inulin and 181.60 g/kg cellulose. *n* = 6 per group. The level of statistical significance was set at *P* < 0.05, and 0.05 < *P* < 0.10 was considered a trend toward significance

In the current study, the bray_curtis distance (Fig. 4A) and UPGMA clustering analysis with bray_curtis distance (Fig. 4B) were used to evaluate similarities in the bacterial communities between the samples. The results showed that the majority of the LF samples formed the first group, while the majority of the HF samples formed the second group, suggesting that the phylogenetic relationship of the LF group was relatively far from the HF group. The principal coordinate analysis (PCoA) profile of bray_curtis distance (Fig. 4C) also revealed that the LF samples dispersed far apart from the HF samples, indicating a clear separation between the two groups. In addition, the analysis of similarities (ANOSIM) test (Fig. 4D) demonstrated that the two groups had significantly different (*R* = 0.304, *P* = 0.009) microbial community structures on day 110 of pregnancy.

**Fig. 5:**
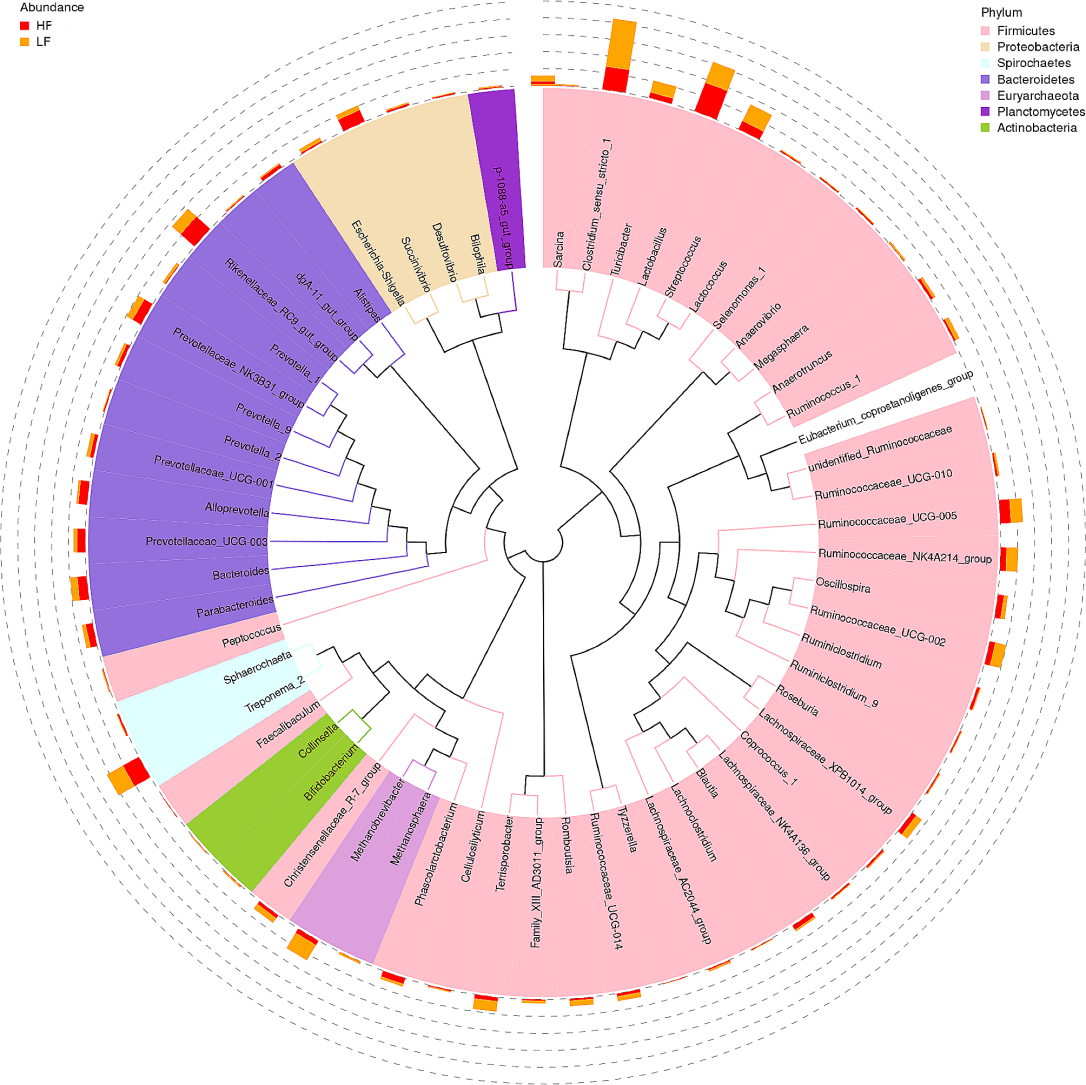
The phylogenetic tree constructed based on the sequence of the top 60 genera. The stacked column chart in the outer circle shows the relative abundance of each genus in different treatments, while the branches with various hues in the inner circle depict their respective phylum. *LF* sows fed a basal diet, *HF* sows fed with the basal diet added with extra 22.60 g/kg inulin and 181.60 g/kg cellulose. *n* = 6 per group

The relative abundances of the fecal microbiota at the phylum (top 10) level are shown in Fig. 4B. Firmicutes and Bacteroidetes were the most predominant phyla in the fecal samples, accounting for 57.3% and 27.4% abundance, respectively. The phylogenetic tree based on the sequences of the top 60 genera (Fig. 5) showed that *Chostridium_sensu_stricto_1*, *Lactobacillus*, *Streptococcus*, and *Treponema_2* were the most abundant genera in the LF group, while *Chostridium_sensu_stricto_1*, *Lactobacillus*, *Treponema_2*, and *Rikenellaceae_RC9_gut_group* were the dominant genera in the HF group.

**Fig. 6:**
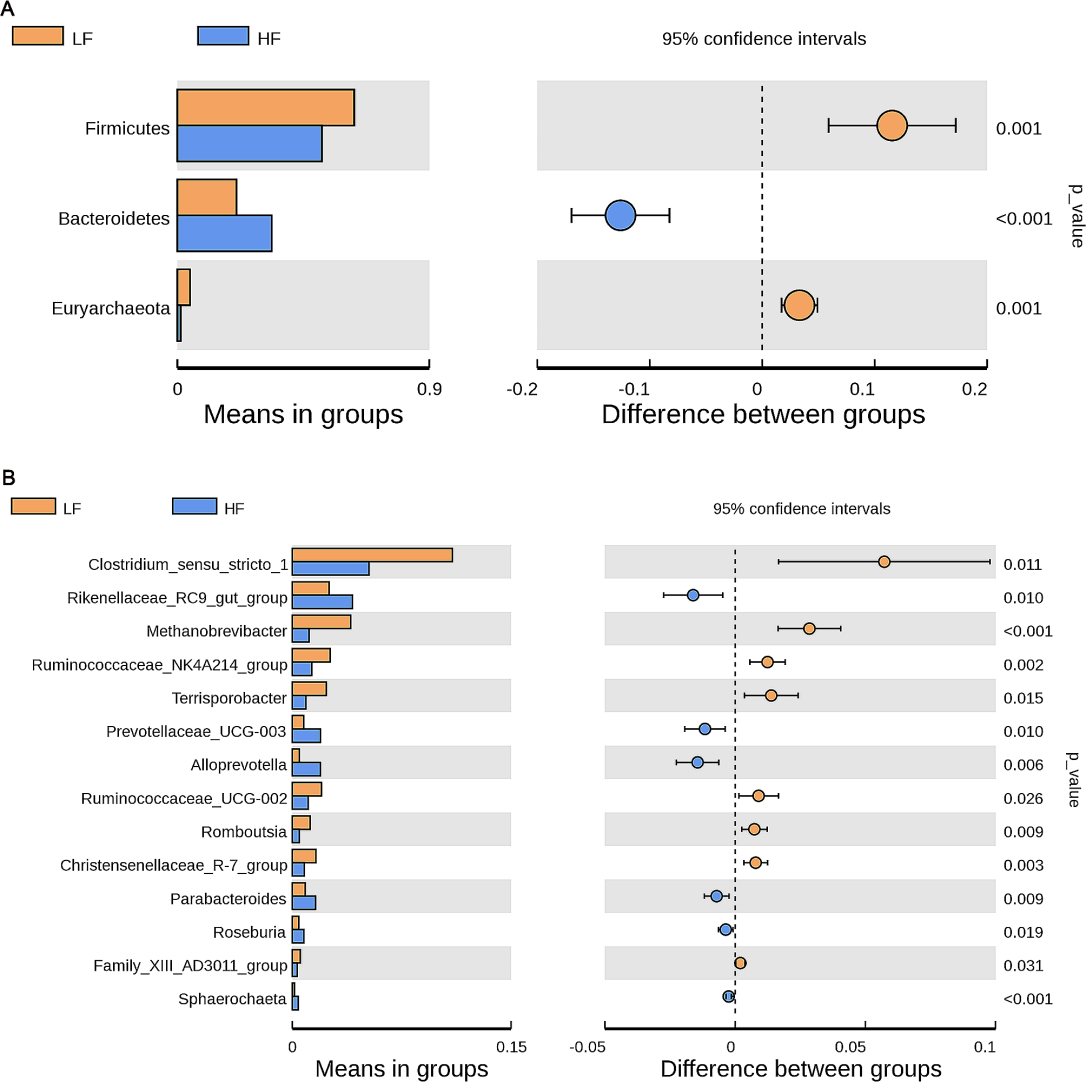
The species of significant differences at phylum (**A**) and genus (**B**) levels. The left picture shows the diversity of species abundance, each of which indicates the mean value of species with significant differences in the abundance, and the right picture shows the difference confidence between the two groups. *LF* sows fed a basal diet, *HF* sows fed with the basal diet added with extra 22.60 g/kg inulin and 181.60 g/kg cellulose. *n* = 6 per group. The level of statistical significance was set at *P* < 0.05

Moreover, the HF group had significantly lower (*P* < 0.05) abundances of Firmicutes and Euryarchaeota and significantly higher (*P* < 0.05) abundance of Bacteroidetes compared to the LF group (Fig. 6A). Among the top 60 genera, the relative abundances of *Chostridium_sensu_stricto_1*, *Methanobrevibacter*, *Ruminococcaceae_NK4A214_group*, *Terrisporobacter*, *Ruminococcaceae_UCG-002*, *Romboutsia*, *Christenellaceae_R-7_group*, and *Family_XIII_AD3011_group* were significantly decreased (*P* < 0.05), while the relative abundances of *Rikenellaceae_RC9_gut_group*, *Prevotellaceae_UCG-003*, *Alloprevotella*, *Parabacteroides*, *Roseburia*, and *Sphaerochaeta* were significantly increased (*P* < 0.05) in the HF group, compared with the LF group (Fig. 6B).

### Correlation analysis between insulin sensitivity, plasma serotonin concentration, and bacterial abundances

**Fig. 7:**
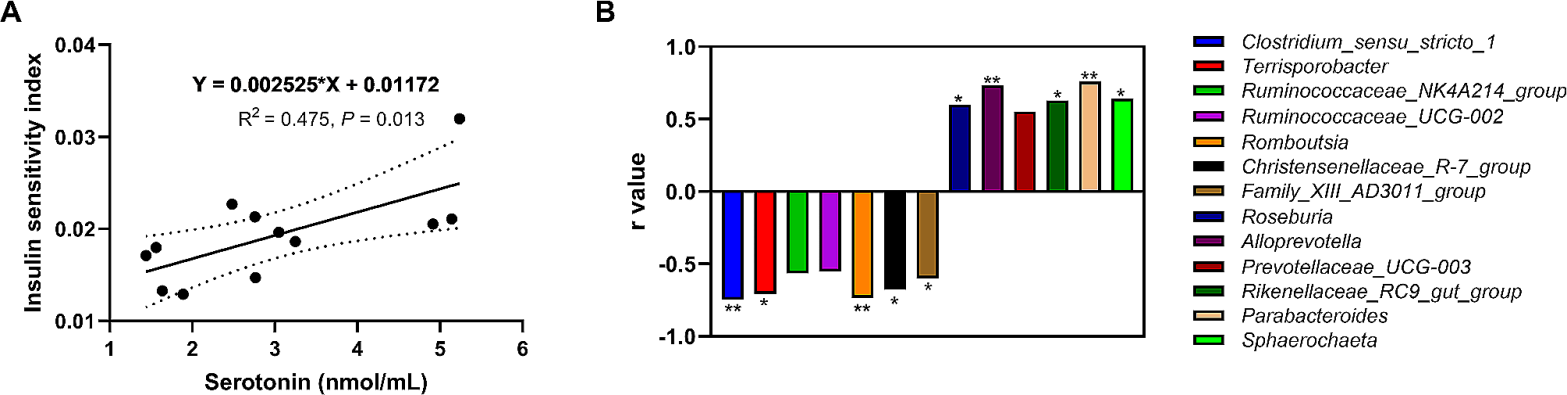
Correlation analysis between insulin sensitivity, plasma serotonin concentration, and bacterial abundances. (**A**) Correlation analysis between insulin sensitivity index and plasma serotonin concentration; (**B**) Correlation analysis between the differential genera and plasma serotonin concentration. *LF* sows fed a basal diet, *HF* sows fed with the basal diet added with extra 22.60 g/kg inulin and 181.60 g/kg cellulose. *n* = 6 per group. The level of statistical significance was set by *P < 0.05 and **P < 0.01

As shown in Fig. 7A, there was a significant positive correlation (*P* < 0.05) between insulin sensitivity and plasma serotonin concentration on day 110 of pregnancy. However, the plasma serotonin concentration was significantly negatively correlated (*P* < 0.05) with the abundances of *Chostridium_sensu_stricto_1*, *Terrisporobacter*, *Romboutsia*, *Christenellaceae_R-7_group*, and *Family_XIII_AD3011_group* and significantly positively correlated (*P* < 0.05) with the abundances of *Roseburia*, *Alloprevotella*, *Rikenellaceae_RC9_gut_group*, *Parabacteroides*, and *Sphaerochaeta* (Fig. 7B).

## Discussion

During late pregnancy, the female body undergoes various physiological and metabolic changes to support the dramatic increase in nutritional needs (particularly glucose requirement) of the fetuses, leading to a decrease in insulin sensitivity [8, 37]. Studies in human and animal models have shown that DF intake improves insulin sensitivity and glucose homeostasis during pregnancy [18, 38]. Sows fed high ISF- or SF-supplemented diets exhibited significantly lower BG concentration in the portal vein, before and after the meal, compared to those fed high starch-supplemented diets [39]. Consistently, our study also indicated that DF consumption decreased FBG in sows during the third trimester. Besides, significantly improved insulin sensitivity index and HOMA-IR by DF consumption were not found on d 85 of pregnancy, but were found on d 97 and 110 of pregnancy in this study. That might because insulin sensitivity decrease began to occur in sows after 85 d of pregnancy [25].

Previous studies have mostly attributed the effect of DF on improving insulin sensitivity to the production of SCFAs via gut microbiota fermentation [19–21]. In this study, we also found that DF consumption promoted the generation of sow fecal SCFAs, including acetate, propionate, butyrate, and total SCFAs. Interestingly, some researches indicated that gut-derived SCFAs could promote the production of colonic serotonin [15, 17]. Serotonin is an end product of tryptophan metabolism, which have been suggested in many diseases, such as diabetes, obesity, inflammatory bowel diseases, etc. [40]. Gut-derived serotonin could be transported into platelets through SERT and released into the bloodstream, to prime the body for energy storage by promoting insulin secretion in the liver and white adipose tissue by interacting with its receptors [41]. A previous study in rats showed that increased peripheral serotonin caused a decrease in circulating hyperglycemia and hyperinsulinemia [42]. The hypoglycemic effect of serotonin may be related to its promotion of glucose utilization and conversion to glycogen [43, 44]. Moreover, serotonin signaling during pregnancy is required for adaptive proliferation of β cells. Kim et al. [45] found that blocking 5-HTR_2B_ signaling inhibited the expansion of maternal insulin-producing beta cells, causing glucose intolerance in pregnant rats. Another study also indicated that serotonin could also act via the 5-HTR3A Na-K-selective ion channel receptor to promote insulin exocytosis, and mice deficient for 5-HTR_3A_ developed glucose intolerance during pregnancy [46]. In this study, DF intake increased preprandial and postprandial serotonin concentrations, which was in accord with Watanabe et al. [12]. Moreover, spearman’s correlation analysis showed that plasma serotonin concentration was positively correlated with the insulin sensitivity index in this study. In general, tryptophan is metabolized via serotonin and kynurenine pathways, resulting in the production of biologically active compounds, such as serotonin, melatonin, and kynurenine [47]. However, increased kynurenine production reduces tryptophan availability for serotonin synthesis. It was reported that increased dietary fiber intake reduced the production of indole [48]. In this study, DF intake increased serotonin/tryptophan ratio, and decreased serum kynurenine level in sows, suggesting that DF consumption promoted serotonin synthesis from tryptophan. Intriguingly, it was reported that systemic serotonin inhibition benefited to improve glucose homeostasis and insulin sensitivity in adipose tissues [49], and one possible reason might be that serotonin inhibited their uptake of glucose from the blood when it acted on tissues other than the liver [42]. Above all, our findings suggesting that DF consumption improved insulin sensitivity during late pregnancy in sows, partially by regulating tryptophan metabolism.

On the other hand, dramatic changed in microbial composition and abundance by DF consumption were found in sow feces in this study. Gut microbiota dysbiosis has been linked to the occurrence of IR in hosts [50]. Numerous studies showed that DF regulated glucose and lipid metabolism by altering the gut microbiota [18, 51]. Moreover, studies have demonstrated that gut microbiota is involved in the regulation of tryptophan metabolism [15, 52]. In the current study, DF consumption increased Simpson and Shannon indexes used to measure community diversity and altered microbial community structures, respectively. It was reported that germ-free mice with low gut microbial diversity showed a decreased production of biologically active serotonin compared with specific pathogen-free mice [52]. The microbial metabolites, such as propionate, butyrate, cholate, and deoxycholate, could promote the release of serotonin from ECs [15]. In the present study, DF consumption during late pregnancy increased abundances of fiber-degrading and SCFA-producing bacteria, including *Rikenellaceae_RC9_gut_group*, *Prevotellaceae_UCG-003*, *Alloprevotella*, *Parabacteroides*, *Roseburia*, and *Sphaerochaeta* in sow feces [53–55]. Furthermore, the abundances of *Rikenellaceae_RC9_gut_group*, *Alloprevotella*, *Parabacteroides*, *Roseburia*, and *Sphaerochaeta* were positively correlated to plasma serotonin concentration. Therefore, our results suggested that DF promoted beneficial microbiome and increased SCFAs which may have impacted the peripheral serotonin level during late pregnancy in sows. However, full-length 16 S rRNA gene amplicon sequencing need be used to reveal the relationship between bacterial flora and serotonin concentration at the species level in the further study.

## Conclusion

Altogether, our results suggest that DF consumption improved insulin sensitivity during late pregnancy in sows, which improved microbial diversity in fecal samples and increased fecal SCFA concentrations, resulting in a positive correlation with plasma serotonin level. Therefore, our findings provide new insights into the regulation of insulin sensitivity in sows.

### Electronic supplementary material

Below is the link to the electronic supplementary material.

Supplementary Material 1

## Data Availability

All sequencing data are deposited in the NCBI Sequence Read Archive database under accession number PRJNA 907161 (Illumina sequences).
