# Supplementary material for: Improvement of insulin sensitivity by dietary fiber consumption during late pregnant sows is associated with gut microbiota regulation of tryptophan metabolism
Source: Anim Microbiome. 2024 Jun 21;6:34. doi: 10.1186/s42523-024-00323-6 (PMC11191243; doi:10.1186/s42523-024-00323-6)
Supplement: Supplementary file 1 — Supplementary Material 1 [file 42523_2024_323_MOESM1_ESM.docx]

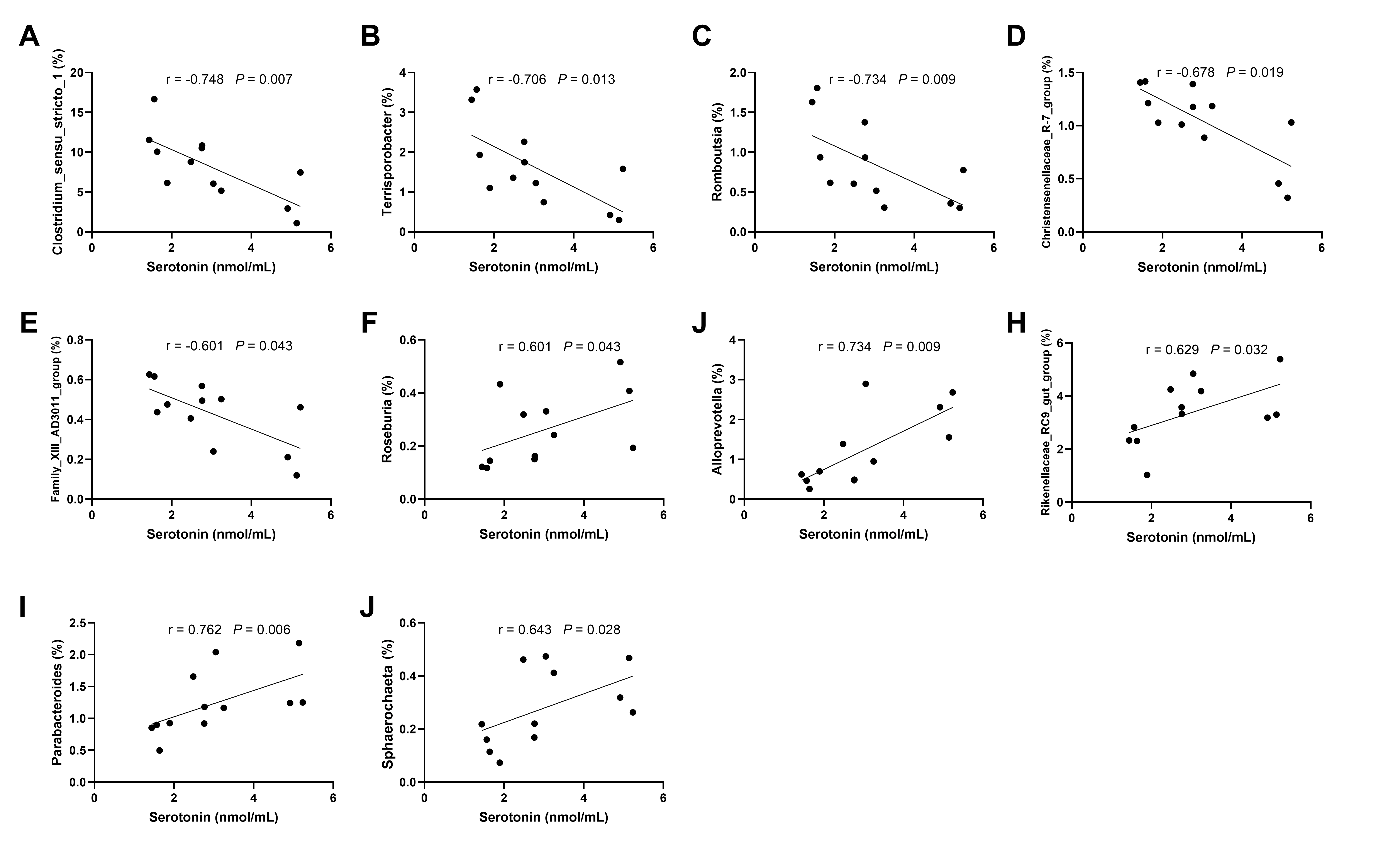


**Figure S1.** Correlation analysis between the differential genera and plasma serotonin concentration. The level of statistical significance was set by *P* < 0.05.
